# Neighborhood socioeconomic characteristics, healthcare spatial access, and emergency department visits for ambulatory care sensitive conditions for elderly

**DOI:** 10.1016/j.pmedr.2018.08.015

**Published:** 2018-09-05

**Authors:** Yuxia Huang, Pamela Meyer, Lei Jin

**Affiliations:** a Department of Computing Sciences, Texas A&M University – Corpus Christi, Corpus Christi, TX 78412, USA; b Department of Psychology and Sociology, Texas A&M University – Corpus Christi, Corpus Christi, TX 78412, USA; c Department of Mathematics and Statistics, Texas A&M University – Corpus Christi, Corpus Christi, TX 78412, USA

**Keywords:** Ambulatory care sensitive conditions, Emergency department visits, Elderly, Spatial access to healthcare, Neighborhood socioeconomic characteristics

## Abstract

The objective of this study is to explore relationships among neighborhood socioeconomic characteristics (for example, income and ethnicity), spatial access to health care, and emergency department (ED) visits for ambulatory care sensitive conditions (ACSC) for adults aged 65 years and over.

ED visit data were from 15 counties in the Texas Coastal Bend from September 1, 2009 and August 1, 2012. ED visits for ACSC that were common for elderly were estimated based on Agency for Healthcare Research and Quality's (AHRQ's) ACSC and Prevention Quality Indicators. The U.S. Census American Community Service (ACS) data provided neighborhood socioeconomic characteristics. Spatial access to general practices and to hospitals, respectively at the zip code level were estimated using the enhanced two-step floating catchment area method. Using multivariable regression models, we estimated associations of elderly ACSC ED visits with neighborhood socioeconomic characteristics and spatial accessibility of healthcare.

We found higher rates of elderly ACSC ED visits are significantly associated with higher rates of elderly Hispanic and poverty at the zip code level. Spatial access to general practices and hospitals play inverse roles in the rate of elderly ACSC ED visits. Poorer access to general practices but easier access to hospitals contributes to the higher elderly ACSC ED rate at the zip code level.

Neighborhood socioeconomic characteristics and spatial access to healthcare affect the rate of elderly ACSC ED visits. Research informing policy action is needed to decrease racial/ethnic and economic disadvantage and increase equitable spatial access to primary care for the elderly.

## Highlights

• Combining effects of neighborhood socioeconomic and spatial access factors on elderly ED visits for ACSC.
• Neighborhood socioeconomics are significantly associated with the rate of elderly ED visits for ACSC at the zip code level.
• Poorer access to general practices contributes to the higher elderly ED rate of ACSC at the zip code level.

## 1 Introduction

Emergency department (ED) visits are costly. A set of medical conditions, such as congestive heart failure, diabetes, and bacterial pneumonia, can be avoidable or reduce the ED visits through timely and effective utilization of primary health care (Enard and Ganelin, 2013). These conditions are often referred to as ambulatory care sensitive conditions (ACSC).

Limiting ACSC ED visits have implications for reducing cost, improving quality, and enhancing efficiency. Weinick et al. (2010) estimated that 13% to 27% of ED visits in the United States could be managed in physicians' offices, clinics, and urgent care centers, saving $4.4 million annually. In 2015, adults aged 65 and older comprised 21.8% of all ED visits (NCHS, 2017). ED ACSC visits may indicate poor access to primary care (Lugo-Palacios and Cairns, 2015; Cecil et al., 2016). Patients who are unable to obtain timely outpatient care often seek care in EDs (Dresden et al., 2015). Studies show that the rate of inpatient hospitalizations for ACSC decreased, while the rate of ED visits for the same conditions increased by 11% from 2008 through 2012 (Fingar et al., 2015). Recent initiatives that penalize hospitals for high readmission rates are leading to more scrutiny over preventable inpatient hospitalization admissions (Fingar et al., 2015), which could lead to the recent decrease in inpatient stays but an increased use of EDs for ACSC (Johnson et al., 2012).

An increasing body of literature aims to understand how neighborhood characteristics (for example, income and ethnicity) influence people's health status. Studies found the neighborhood disadvantage were associated with transportation availability for health seeking (Rachele et al., 2017a), body mass indices (Rachele et al., 2017b), and ACSC ED visits (Fishman et al., 2018; Lugo-Palacios and Cairns, 2015).

Barriers to health care can be grouped into five dimensions: availability, accessibility, affordability, acceptability, and accommodation (Penchansky and Thomas, 1981). The first two are spatial, where availability refers to the number of local facilities that a patient can choose and accessibility is the travel impedance (distance or time) between patient and facility locations. Spatial accessibility of healthcare considers these two factors simultaneously (Guagliardo, 2004). The last three are non-spatial and reflect healthcare financing arrangements and cultural factors.

The effects of the aspatial barriers on ED visits are well documented (Jaeger et al., 2015; Gasperini et al., 2017). However, relatively few studies examine their relationships with spatial factors (Chen et al., 2015; Fishman et al., 2018). Furthermore, research demonstrated that elderly living in the poor areas had significantly higher chances of having ACSC (Menec et al., 2010). Although neighborhood socioeconomic characteristics are associated with ACSC ED visits (Fishman et al., 2018), their combined effects with spatial factors are not fully understood.

The objective of this study is to explore the relationships among neighborhood socioeconomic characteristics, spatial access to health care, and elderly ACSC ED visits, expanding Fishman's study (Fishman et al., 2018) by focusing on adults aged 65 and older for a large regional area. In this paper, in addition to examining primary care facilities that include family and general practitioners and general internists (Starfield et al., 2005), we examine hospitals because the distribution and setting of hospitals may affect the utilization of elderly ACSC ED (Chen et al., 2015). We hypothesize that:

1. Neighborhood disadvantages are associated with the rates of elderly ACSC ED visits.
2. Areas with poorer access to both general practices and hospitals are more likely to have higher rates of elderly ACSC ED rates than those with easier access.

## 2 Methods

This is a retrospective analysis using a complete dataset of ED visits from 15 counties in the Texas Coastal Bend. In 2010, this area had over 32,000 Hispanic older adults, accounting for over 40% of the entire senior population. Since 2010, three health needs assessments indicated limited access to healthcare services in this area, particularly for older adults in terms of the three non-spatial barriers, including affordability, acceptability and accommodation (Meyer et al., 2016).

### 2.1 Data sources

We obtained a complete dataset of ED visits from all local hospital systems serving the Coastal Bend area, from September 1, 2009 to August 31, 2012, including all outpatient encounters in all licensed emergency departments and ambulatory surgical centers in the area. To protect privacy, the patient's home address is not given, instead each ED admission includes the patient's county, residence zip code, age, discharge date, primary diagnosis, secondary diagnosis codes, etc. A person who is admitted to the ED multiple times during the study period is counted each time as a separate “discharge” from the ED. For the analysis, ACSC ED visits were aggregated at the zip code level, the smallest available spatial unit in the ED dataset, following the methods used in health studies (Starfield et al., 2005). The analysis included all zip codes that contained at least one older adult, which resulted in a total of 74 zip codes (32 urban and 43 rural/small town). The median size of the zip codes in the urban area is 27 km^2^ while in the rural/small town area the median size is 228 km^2^. In the urban areas, the median elderly senior population and elderly Hispanic population were 1087 and 344, respectively; whereas in the rural/small town areas, the median elderly senior population and elderly Hispanic population were 284 and 133, respectively.

To be consistent with the ED visit data, we used the zip code level where patients resided to represent neighborhood socioeconomic characteristics. The characteristics include the following six factors and all are for the population 65 years and over: percent elderly Hispanic (*Hispanic*), percent elderly population with less than a high school education (*Education*), percent elderly population below poverty level (*Poverty*), percent elderly disability status by health insurance coverage status (*Disability*), percent elderly living alone elderly population (*Living alone*), and percent elderly population who speak Spanish (*Language*). To calculate each percentage, the numerator, which is the corresponding population in a zip code, was obtained from US Census American Community Study (ACS) 2008–2012 summarized data. The denominator of the percentage is the total senior population at the zip code.

The individual-level data on healthcare facilities was obtained from the business database InfoUSA, the US's largest business list company. Primary healthcare (general practices) are under standard industrial classification (SIC) code 801 but we excluded those not for senior patients. Hospitals included all businesses under SIC code 806 that include hospitals, emergency medical and surgical services. A total of 476 primary healthcare facilities and 36 hospitals were identified in the study area.

### 2.2 Procedures

According to the local hospital systems, there were a total of 749,780 ED visits during the study period. We first excluded the visits that were not in the 15 counties based on patient's county. We then extracted the elderly visits based on patient's age. As a result, we identified a total of 66,878 ED visits for elderly (65 and older) who lived in the 15 counties. After that, we estimated the number of ACSC ED visits based on the Agency for Healthcare Research and Quality (AHRQ)'s ACSCs and Prevention Quality Indicators (PQIs). In this study, we considered all the conditions that have at least 150 admissions (0.2% of all ED visits) and are common ACSCs for elderly. Overall >60 conditions were included and the list is provided in Table 1. Of the ED visits for elderly, there were 13,686 for ACSC. The rate of ACSC ED visits at each zip code was then calculated by the number of ACSC visits divided by the total elderly population at the zip code level. The data presented here are observed visits and are not risk-adjusted for age or gender. The Institutional Review Board (IRB) approved the project and the presentation of these data is compatible with HIPPA requirements.

**Table 1:** Summary of common ACSC ED visits for elderly (65 and older), September 1, 2009–August 31, 2012.

Condition	ICD-9-CM Code(s) (Primary diagnosis)	Common ACSC ED Visits	
All conditions		n = 13,686	%
Chronic conditions		6544	47.8%
Asthma	493 (493.*)	485	3.5%
Grand mal and other epileptic convulsions	345, 780.3	252	1.8%
Cellulitis	681, 682, 683, 686	1184	8.6%
Diabetes	250.1, 250.2, 250.3, 250.8, 250.9, 250.0	952	7.0%
Congestive heart failure	428, 402.01,402.11,402.91,518.4	844	6.2%
Chronic obstructive pulmonary disease	491,492,494,496,466.0 [466.0] only with secondary diagnosis of 491,492,494 and 496	1278	9.3%
Hypertension	401.0, 401.9, 402.00, 402.10, 402.9	1549	11.3%
Acute conditions		7142	52.1%
Angina	411.1, 411.8, 413	165	1.2%
Gastroenteritis, dehydration, hypoglycemia	558.9, 276.5, 251.2	1501	11.0%
Bacterial pneumonia	481,482.2, 482.3, 482.9, 483, 485, 486	746	5.5%
Kidney/urinary infections	590, 599.0, 599.9	3898	28.5%
Severe ear, nose and throat infections	382,462,463,465,472.1	832	6.1%

### 2.3 Measurement of spatial accessibility of healthcare

We adopted the enhanced two-step floating catchment area (E2SFCA) method (Luo and Qi, 2009) to measure spatial access to general practices and to hospitals, respectively at the zip code level. The measurement consisted of two steps. The first step was to calculate the physician-to-population ratio for each facility, which is the ratio of the capacity of the facility over the total population within the maximum catchment area of the facility. The number of physicians at each facility represents the facility's capacity (Luo and Qi, 2009; Luo and Wang, 2003). Traveling time within the street network represents the catchment area, the area that a facility can provide the service. Following most existing work, a thirty minutes driving zone within street network was used as the maximum catchment for general practices (Luo and Wang, 2003; Shi et al., 2012) and the maximum catchment of hospitals was defined as within 60 min driving zone because the golden hour is a common standard (McGrail and Humphreys, 2009). The second step was to measure the accessibility value for each zip code through summing up the physician-to-population ratio (calculated in the previous step), of all facilities that are within the maximum area catchment area of the zip code. For the maximum catchment for population areas, 30 min driving zone for primary health facilities and 60 min for hospitals were applied to urban/metropolitan areas. In small town or rural areas, however, the size of maximum catchments was doubled in order to include some isolated areas in the analysis (McGrail and Humphreys, 2009). The urban and rural areas were determined using 2012 Rural Urban Commuting Area codes (RUCA, 2017). Distance decay was applied in both steps, which means a facility is more likely to provide service to people who live nearby than those who live far away. Similarly, a person is more likely to visit a closer facility than those that are far away. In our study, similar to most studies in measuring spatial access to health care facilities, there is no decay within the first 10 min. However, in the areas between 10 min and the maximum catchment, we adopted a widely used continuous decay function by McGrail and Humphreys (2009).

### 2.4 Statistical analysis

As stated in Section 2.1, the six neighborhood socioeconomic factors include *Hispanic*, *Education*, *Poverty*, *Disability*, *Living alone* and *Language*. Because some factors are correlated, we first used the effect screening process provided by statistical software JMP Pro 13 to identify the key factors that have the greatest effect on the rate of ACSC ED visits. The effect screening platform uses the principle of effect scarcity (Box and Meyer, 1986) to guide users in determining which factors are significant to the response. The identified key factors were then used to examine their relationships with the rate of ACSC ED visit rate using ordinary least squares (OLS). To obtain a better understanding, we first only used the identified key socioeconomic factors from the effect screening (Model 1) and then controlled for the two spatial accessibilities (Model 2), one is of general practices and the other is of hospitals. The two spatial access variables were log transformed using −ln(x) for two reasons. The first is to be consistent with other variables. For spatial accessibility values, a higher score represents a better spatial accessibility while a lower score represents a poorer accessibility. The second reason is to approximate percent changes of the two spatial accessibility variables. The unit of neighborhood variables is ratio. Spatial accessibility values, however, are the actually measured accessibility values for each zip code, and they have very small values compared with other variables. The OLS analysis was conducted using ESRI ArcInfo 10.4.1.

## 3 Results

Overall, 20.5% of ED visits by elderly patients had a primary ACSC diagnosis during the study period (Table 1). The most three common primary diagnoses were kidney/urinary infections (28.5%), hypertension (11.3%), and gastroenteritis/dehydration/hypoglycemia (11%). Table 2 displays the significant differences in characteristics of those with ACSC compared to those with non-ACSC ED visits. Compared with non-ACSC visits, the probability of an ACSC visit to the ED was higher for older adults who were 80 years and over, female, Hispanic, and unemployed.

**Table 2:** Characteristics of elderly (over 65 years) with common ACSC ED visits, September 1, 2009–August 31, 2012.

		ED Visits					
		All Visits		ACSC		Non-ACSC	
		n = 66,878	%	n = 13,686	%	n = 53,192	%
Age	≥ 80	23,328	34.9%	4848	35.4%	18,480	34.7%
	<79	43,550	65.1%	8838	64.6%	34,712	65.3%
Gender	Female	40,939	61.2%	8848	64.7%	32,091	60.3%
	Male	25,939	38.8%	4838	35.3%	21,101	39.7%
Race	White	15,173	22.7%	2966	21.7%	12,207	22.9%
	Hispanic	17,844	26.7%	3724	27.2%	14,120	26.5%
	Others	2227	3.3%	437	3.2%	1790	3.4%
	Unknown	31,634	47.3%	6559	47.9%	25,075	47.1%
Occupation	Retired	50,180	75%	10,363	75.7%	39,817	74.9%
	Unemployed	9842	14.7%	2155	15.7%	7687	14.5%
	Others	6856	10.3%	1168	8.5%	5688	10.7%

Table 3 presents the results of two multivariate OLS regression models. The top three key neighborhood socioeconomic factors identified from the screening process include *Hispanics*, *Disability*, and *Poverty*. These three variables together with the two spatial accessibilities (namely spatial accessibility of general practices and of hospitals) were the explanatory variables while the rate of ACSC ED visits is the dependent variable in the OLS regression.

**Table 3:** Ordinary least squares (OLS) analysis: association effects of spatial accessibility and neighborhood socioeconomic characteristics on common ACSC ED visits for elderly (65 and over).

	The rate of ACSC ED visits for elderly							
	Model 1				Model 2			
Characteristic	Coeff	Std. error	Robust_Pr	VIF	Coeff	Std. error	Robust_Pr	VIF
Socioeconomic								
Hispanics	0.139	0.051	0.003^⁎⁎⁎^	1.506	0.101	0.054	0.051^⁎^	1.722
Poverty	0.310	0.114	0.040^⁎⁎^	1.428	0.306	0.114	0.029^⁎⁎^	1.465
Disability	−0.123	0.084	0.227	1.137	−0.130	0.084	0.235	1.187
Spatial accessibility								
General practices	…	…	…	…	0.033	0.016	0.053^⁎^	4.347
Hospitals	…	…	…	…	−0.043	0.022	0.050^⁎⁎^	4.215
R^2^	0.22				0.24			

^⁎^ An asterisk next to a number indicates a statistically significant p-value (p < 0.01): ^⁎^ p < 0.1, ^⁎⁎^ p < 0.05, ^⁎⁎⁎^ p < 0.01, ^⁎⁎⁎⁎^ p< 0.001

The large variance inflation factor (VIF) value for each variable in all models is <7.5, indicating no redundancy among explanatory variables in both models. Since the Koenker (BP) Statistics are statistically significant in most models, the p-value of the robust test (Robust_Pr) is used to determine coefficient significance. The values of Moran's *I*, the method used to examine the clustering on the residuals, are 0.045, and 0.047, respectively for Models 1 to 2, indicating no clustering or significant patterns in the residuals of the data fitting using both models.

By only considering the neighborhood socioeconomic variables themselves, both *Hispanics* and *Poverty* are significantly positively associated with the rate of ACSC ED visits (Model 1): the higher elderly Hispanics rate or poverty rate, the higher the rate of ACSC ED visits at the zip code level. The association between *Disability* and the rate of ACSC ED also positive, but not statistically significant.

After controlling simultaneously for neighborhood socioeconomic and spatial variables, the relationship between the rate of ACSC ED visits (Model 2), the performance of the rate of ED visits was slightly increased with an increase of R^2^ from 0.22 (Model 1) to 0.24 (model 2). The socioeconomic variables persisted although they are attenuated. Both spatial accessibility of general practices and spatial accessibility of hospitals are significant for the rate of ACSC ED visits. However, surprisingly, we observed a positive relationship between the rate of ACSC ED visits and spatial accessibility of general practices while this relationship is negative for spatial accessibility of hospitals: the poorer access to primary care or the better access to hospitals, the higher the rate of ACSC ED visits by elderly.

## 4 Discussion

This study is one of a few (Fishman et al., 2018) to combine effects of neighborhood socioeconomic and spatial accessibility factors on the rate of ED visits for common ACSC and the only one to focus on elderly adults. We demonstrated the associations among neighborhood socioeconomic characteristics, spatial access to healthcare, and the rate of elderly ACSC ED visits at the zip code level. As we expected, our findings show that higher rates of elderly ACSC ED visits are significantly associated with higher rates of elderly Hispanics and elderly poverty. However, we found that spatial accesses to general practices and hospitals play opposite roles in the rate of elderly ACSC ED visits. Poorer access to general practices to hospitals contributes to the higher elderly ACSC ED rate. Surprisingly, areas with easier access to hospitals are more likely to have higher rates of elderly ACSC ED rates than are those with poorer access.

We observed that neighborhood socioeconomic disadvantages are significantly associated with rates of elderly ED visits for common ACSC, even after controlling for spatial accessibility of healthcare. Social inequalities in access to health care persist in the U.S. health care delivery system and lower-income residents are less likely to receive preventive services and more likely to experience delays in their care (Chang and Pope, 2009; Reyes and Hardy, 2015). This study confirms that those who are in poverty often lack a medical home and are more likely to use ED for primary care once the episode becomes acute (Jiang et al., 2014). Elderly who become eligible for Medicare, compared to their counterparts who have regular access to health insurance, may have declining health and seek care only when in a crisis.

Similar to Lin's results (Lin et al., 2016), our study finds that areas with higher proportion of senior Hispanics rates are significantly associated with elderly ACSC ED visits. In the Coastal Bend, we found that Hispanic and White adult patients do not follow the same pattern of spatial distribution (Huang and Meyer, 2012). One possible explanation is that seniors of lower-income areas are more likely than seniors of the wealthiest areas to use ED for ASCS visits. In the U.S., the elderly disabled Hispanics are more likely to be in poverty than their non-Hispanic counterparts (He and Larsen, 2014). Previous studies show that class and racial differences exist in rates of potentially avoidable hospitalization and the rates are significantly higher among lower-income populations (Jiang et al., 2014).

Our findings show a negative association between elderly disability and the rate of elderly ACSC ED visits. Seniors living in areas with high percentage of elderly disability rates are less likely than other areas to seek ED for ACSC. We suspect that this reflects the living arrangements of the elderly. Disabled elderly is defined as those individuals who require assistance with basic Activities of Daily Living (ADL) (Burwell and Jackson, 1994). In the U.S., 73.5% of elderly Hispanics live with others compared to 59.9% of elderly Non-Hispanics (He and Larsen, 2014). Older Mexican Americans who suffer from multiple chronic conditions such as diabetes and heart disease had more mobility and ADL limitations (Collins et al., 2018). In Texas, 65.7% of elderly with a disability live with others (He and Larsen, 2014). Living with others may prevent inpatient hospitalizations as care givers may intercept before the seniors' condition declines and requires hospitalization. Disabled elderly living in nursing homes also may find that institutional care givers may avert hospitalizations.

The negative relationship between spatial access to primary care and the rate of elderly ACSC ED visits is consistent with the previous studies (Lugo-Palacios and Cairns, 2015; Cecil et al., 2016). Better access to and use of primary care services (Jaeger et al., 2015) or clinics (Fishman et al., 2018) could reduce health care costs and relieve ED overcrowding. We found a positive relationship between spatial access to hospitals and the rate of elderly ACSC ED visits, indicating that easy access to hospitals could increase the rate of elderly ACSC ED visits. Others found that Medicare and Medicaid patients (Chen et al., 2015) or racial/ethnic minorities (Fishman et al., 2018) who live closer to the hospital ED have higher probability for non-emergent visits than other patients. One possible explanation is that elderly who live in areas with less spatial access to primary healthcare facilities will seek help only when in a crisis resulting in ED visits for preventable conditions. This issue requires further research.

## 5 Conclusion

This study demonstrated the effects of neighborhood socioeconomic characteristic and spatial accessibility of health care on the rate of elderly ACSC ED visits. The study showed that spatial access to primary care and spatial access to hospitals play different roles in the rate of ED visits by older adults for ACSC. Strengths of this study include utilizing all ED data in a well-sized region, linking spatial factors to socioeconomic attributes to the rate of ED visits for elderly potentially preventable conditions.

Our study has several limitations that merit discussion. First, it is possible that data limitations biased the results. Similar to other research that used ED admission data, a major limitation is that we could not determine how many ED visits were by the same patient due to data confidentiality laws. Neighborhood and social determinants may impact repeat use of EDs, thus affecting our results as to who comes from where for health care. Travel distance between home to ED might be a much greater barrier if one has no transportation. Due to the data availability, we could not tell whether the patient arrived to the ED by ambulance or from a skilled nursing facility. Second, the results are based on 15 counties in Texas, which might affect the generalizability of the findings at a national level. The results, however, are consistent with other national studies assessing ACSC ED usage. Third, AHRQ's ACSC and PQIs are designed for hospital discharge. Selection bias may exist in identifying ACSC ED visits. Last, the neighborhood socioeconomics in this study describe the population at the zip code level, which is the smallest spatial unit from the ED visits data. In addition to the population of elders and Hispanic elders, other characteristics such as individual level socioeconomic data would allow more specific analyses, such as examining similar variability across the zip codes in the future.

Disparities in access to primary health care exist for U.S. elderly, even for those with Medicare insurance. Neighborhood socioeconomic disadvantages are associated with rates of elderly preventable ED visits. These situations are costly, both for people's health status and economically, for local and national health care systems. Research into strategies that increase access to primary health care for elderly living in socioeconomically neighborhoods is warranted. Health providers and policy makers should work to increase equitable access to primary health care in order to decrease potentially preventable ED usage. This could improve the number of elderly with medical homes that would help increase equitable distribution of health in populations.
